# Macrophages and angiogenesis in human lymphomas

**DOI:** 10.1007/s10238-023-01291-y

**Published:** 2024-01-29

**Authors:** Domenico Ribatti, Roberto Tamma, Tiziana Annese, Giuseppe Ingravallo, Giorgina Specchia

**Affiliations:** 1 Department of Translational Biomedicine and Neuroscience, University of Bari Medical School, Bari, Italy; 2 Department of Medicine and Surgery, Libera Università del Mediterraneo (LUM) Giuseppe Degennaro University, Bari, Italy; 3 Section of Pathology, Department of Precision and Regenerative Medicine and Ionian Area, University of Bari Medical School, Bari, Italy; 4 School of Medicine, University of Bari Medical School, Bari, Italy

**Keywords:** Angiogenesis, Anti-angiogenesis, Lymphoma, Tumor-associated macrophages

## Abstract

A link exists between chronic inflammation and cancer and immune cells, angiogenesis, and tumor progression. In hematologic malignancies, tumor-associated macrophages (TAMs) are a significant part of the tumor microenvironment. Macrophages are classified into M1/classically activated and M2/alternatively activated. In tumors, TAMs are mainly constituted by M2 subtype, which promotes angiogenesis, lymphangiogenesis, repair, and remodeling, suppressing adaptive immunity, increasing tumor cell proliferation, drug resistance, histological malignancy, and poor clinical prognosis. The aim of our review article is to define the role of TAMs and their relationship with the angiogenesis in patients with lymphoma reporting both an analysis of main published data and those emerging from our studies. Finally, we have discussed the anti-angiogenic approach in the treatment of lymphomas.

## Introduction

The link between chronic inflammation and cancer was first proposed by Rudolf Virchow in 1863, establishing a relationship between inflammatory cell infiltration and cancer [1]. Moreover, inflammatory cells in tumor microenvironment contribute to tumor angiogenesis through the release of several angiogenic factors [2, 3]. Macrophages recruited to the tumor microenvironment are the tumor-associated macrophages (TAMs). TAMs can reach up to 50% of the total tumor mass and are concentrated at the invasive edge of the tumor [4, 5]. Different chemokines and growth factors, including colony-stimulating factor(CSF)-1, monocyte chemoattractant protein (MCP)-1/CCL-2, CCL-3, CCL-4, CCL-5, CCL-7, CCL-8, CXCL-12, interleukin (IL)-10, vascular endothelial growth factor (VEGF), and platelet derived growth factor (PDGF), secreted by tumor or stromal cells, recruit blood circulating monocytes which then differentiate into TAMs in the tumor site [6]. TAMs are classified into M1/classically activated and M2/alternatively activated [5]. Hypoxia-inducible factor-1 (HIF-1) regulates the immune response, viability, migration, phenotypic plasticity, and metabolism of macrophages [4]. Specifically, HIF-1*α* promotes macrophage M1 polarization by targeting glucose metabolism. Moreover, the HIF/VEGF axis, as well as the release of other mediators, is involved in the amplification of tumor angiogenesis [5]. In this context, TAMs release both pro-angiogenic and angiostatic factors (Table 1). M1 is the pro-inflammatory subtype through the section of molecules with pro-inflammatory activity (Table 1) promoting Th1 T -cells and is activated by granulocyte macrophage colony-stimulating factor (GM-CSF), interferon gamma (IFN*γ*), and bacterial products. M2 is the pro-tumoral subtype promoting Th2 T cells, angiogenesis, and immunosuppression through the release of anti-inflammatory molecules (Table 1). The phenotype of M1–M2 macrophages may be reversed [7, 8]. CD163 is a phenotypic marker of M2 macrophages that can be used to distinguish M2 and M1 macrophages, while CD68 is a pan-macrophage marker, and its expression is upregulated in M1 compared to M2 macrophages.

**Table 1:** Pro-angiogenic and angiostatic factors released by tumor-associated macrophages (TAMs). Pro-inflammatory molecules secreted by M1-TAMs and anti-inflammatory molecules expressed or released by M2-TAMs

Pro-angiogenic factors	FGF-2 VEGF IL-6 IL-8 PDGF G-CSF GM-CSF
Angiostatic factors	TSP-1 IL-12 IL-18 CXCL-9 CXCL-10
Pro-inflammatory molecules	IL-1 IL-6 IL-12 IL-23 TNF*α* Nitric oxide CXCL-9 CXCL-10 CXCL-11
Anti-inflammatory molecules	IL-10 TGF*β* CCL-17 CCL-18 CCL-22 Class A scavenger receptor (CD204) Mannose receptor C type 1 (CD206) Hemoglobin scavenger receptor (CD163)

s

MiR-126 suppresses the recruitment of inflammatory monocytes into the tumor stroma [9], while overexpression of hypoxia-inducible miR-210 increases the recruitment of monocytes and their M2 macrophage [10]. Other mechanisms are involved in the recruitment of TAMs in the tumor microenvironment, including chemoattractants and their receptors, such as chemokine ligand (CCL) 2/CC receptor (R)2^+^, CCL-2/CCR-5^+^, IL-1*β*/IL-1 receptor (R), VEGFA/VEGFR, CSF1/CSF R, tyrosine-protein kinase receptor (Tie)/angiopoitein 2 (Ang2), CCL-5, C-X-C motif chemokine ligand (CXCL) 10, CXCL-12, and complement C1q, the latter is the most potent attractant promoting M2-like TAMs recruitment [11]. Tumor cells produce factors that promote TAMs recruitment, which, in turn, secrete different cytokines and express their receptors, allowing their own recruitment [12].

In tumors, TAMs are mainly constituted by M2 subtype [13], which promotes angiogenesis, lymphangiogenesis, repair, and remodeling, suppressing adaptive immunity, increasing tumor cell proliferation, drug resistance, histological malignancy, and poor clinical prognosis [14]. M2 macrophages express high levels of IL-10, low levels of IL-12 and IL-13 [15], and contribute to immune suppression through IL-10 and transforming growth factor beta (TGF*β*) [16]. In regressing and non-progressing tumors, TAMs mainly resemble the M1 type and exhibit anti-tumor activity, while in malignant and advanced tumors, TAMs are biased toward the M2 phenotype that favors tumor malignancy [17].

## Human lymphomas

Non-Hodgkin lymphomas (NHLs) include neoplasms infiltrating various lymphoid structures which may arise from B lymphocytes, T lymphocytes, and natural killer (NK) cells and are characterized by a tendency to disseminate toward extra-nodal locations [18]. About 25% of NHLs arise in extra-nodal locations and most of them are present in both nodal and extra-nodal sites. Based on their morphology, immunophenotype, genetic, and clinical features, NHLs have been classified into more than 30 different types [19]. Histological features allow to discriminate between a nodular and a diffuse pattern. In the nodular pattern, the tumor cells aggregate to form large clusters, while the diffuse is characterized by an impairment of lymph node architecture [20]. Clinical trials allowed us to distinguish the B cell lymphoma histological subtypes as indolent, aggressive, and very aggressive based on their typical clinical behavior [21, 22]. The indolent lymphomas, whose overall survival is measured in years [23], represent about 40 percent of NHL and include follicular lymphomas (FL), chronic lymphocytic leukemia/small lymphocytic lymphomas (CLL/SLL), a fraction of mantle cell lymphomas (MCL) cases, extra-medullary, nodal, and splenic marginal zone lymphomas (MZL), and lymphoplasmacytic lymphomas (LPL). The aggressive group includes large B cell lymphomas, subdivided into anaplastic and primary mediastinal lymphomas, and various kinds of diffuse large B cell lymphomas (DLBCL). In this group, untreated patients survive a few months, even if treatment may lead to definitive remissions and cure in a significant number of patients [24]. The highly aggressive group is characterized by survival of a few weeks if not adequately treated. The elected therapies for B cell NHL are chemotherapy, radiotherapy, and immunotherapy, used either as monotherapies or as combined therapies [25]. Classical Hodgkin’s lymphoma (CHL) is one of the most common lymphomas characterized by the presence of large multi- and mono-nucleated cells, Reed–Sternberg (RS) and Hodgkin (H) cells, respectively [26]. RS and H cells correspond to 1–10% of total tumor mass; the remaining 90% is composed of tumor inflammatory cells, including T and B lymphocytes, plasma cells, histiocytes/macrophages, granulocytes, eosinophils, mast cells, and mesenchymal stromal cells (MSCs) [27]. How RS cells interact with the tumor microenvironment is still a debated question. RS and H cells carry Ig genes somatic hypermutations and clonal Ig rearrangements, suggesting their origin from pre-apoptotic germinal center (GC) B cells [28]. RS cells present an unusual immunophenotype characterized by the absence of B cell markers, associated with possible co-expression of molecules of various hematopoietic lineages [29]. RS cells induce programmed death ligand-1 (PDL-1) expression in macrophages [30] and induce M2 phenotype in vitro [31]. The number of TAMs is higher in EBV-related CHL [32]. TAMs faster FL growth and survival via the CD40 axis [33] and can activate the B cell receptor [34]. In FL, it has been demonstrated an increased number of PDL-1-expressing TAMs [35].

## Macrophages and angiogenesis in non-Hodgkin lymphomas (Table 2)

The tumor microenvironment constitutes about half of the tumor mass in indolent FL and marginal zone lymphoma, whereas the proportion in aggressive DLBCL is generally lower and scarce in Burkitt’s lymphoma [36, 37]**.** TAMs found in DLBCL and correlate with a poor prognosis [38–41]. Pro-angiogenic M2 TAMs were found in FL and DLBCL through the secretion of angiogenic factors and matrix metalloproteinases (MMPs) which remodel the extracellular matrix [42, 43]. In MCL, TAMs expression was positively associated with ki67 proliferative index and negatively associated with overall survival [44]. Moreover, in MCL infiltration of CD163^+^ cells adversely affect outcome independent of established risk factors [45]. VEGF-A levels in the serum of patients with progressive NHL were significantly elevated in comparison with patients with complete remission [46, 47]. Elevated VEGF-A levels have been found in aggressive B cell lymphoma subtypes, including MCL, DLBCL, and CLL and small lymphocytic lymphoma [48–51].

DLBCLs have been described a “stromal 1” signature associated with a favorable prognosis and include the expression of MMP-2 and MMP-9, tissue inhibitor of matrix metalloproteinase-2 (TIMP-2), and a “stromal 2” signature is associated with a poor clinical outcome and is found in tumors with a high microvascular density [52].

## Macrophages and angiogenesis in Hodgkin lymphomas (Table 2)

**Table 2:** Tumor-associated macrophages (TAMs) in different types of lymphomas (references between brackets)

Non-Hodgkin lymphoma	
Diffuse large B cell lymphoma (DLBCL)	M2 TAMs positively correlate with a poor prognosis [38, 39]
Follicular lymphoma	M2 TAMs number correlates with angiogenesis [42]
Mantle cell lymphoma	M1 and M2 TAMs positively correlate with ki67 and negatively with overall survival (OS) [50] M2 TAMs adversely affect outcome [73]
Classic Hodgkin lymphoma	TAMs involved in the mechanism of action of checkpoint inhibitors [35] TAM-derived PDL-1 and HRS cell-derived PD-L1 and PD-L2 neutralize the activity of PD-1^+^ T cells and NK cells [55] Higher number of CD68^+^ TAMs is associated with shortened survival and the outcome of autologous stem-cell transplantation [64] M1 TAMs number correlates with favorable prognosis in the mixed cellularity cHL [69] Lack of TAMs is beneficial for HL growth [76] High number of CD163^+^ TAMs correlates with VEGF-A levels and increased microvascular density [77]

TAMs are present in cHL [53, 54]. In the tumor microenvironment, PD-L1^+^TAMs, and programmed cell death protein-1 (PD-1)^+^ CD4^+^ T cells are present, in contact with PD-L1^+^ tumor cells, supporting a possible role of the TAMs in the mechanism of action of checkpoint inhibitor therapy [30]. TAM-derived PDL-1 in conjunction with the HRS cell-derived PD-1 ligands PD-L1 and PD-L2 neutralizes the anticancer activity of PD-1^+^ T cells and natural killer (NK) cells, that can be reversed utilizing PD-1 blocking antibodies [55]. A higher number of CD68^+^ TAMs was associated with shortened survival and with the outcome of autologous stem-cell transplantation [56]. Other studies have confirmed the relationship between TAMs and lower outcomes after upfront treatment [57–60]. The molecular characterization of HR cells reported as neoplastic clones the overexpression of CSF1 receptor (CSF1R), a gene of the macrophage signature, and the latter gene was associated with primary treatment failure [61]. A correlation between the number of M1 TAMs and favorable prognosis in the mixed cellularity subtype of cHL has been reported [62]. PI3K activity is essential for M2 polarization [63].Dual PI3K/inhibition suppresses M2 macrophage polarization in HL through PKM downregulation [64]. A lack of TAMs is beneficial for HL growth, while TAMs have an inhibitory effect with an increasing number [65].

A high number of CD163 ^+^ TAMs correlate with elevated VEGF-A levels and an increased microvascular density [66].

## Personal experience (Table 3)

**Table 3:** Personal experience (references between brackets)

Diffuse large B cell lymphoma (DLBCL)	Higher number of CD68^+^ TAMs and microvessels in the non-responder versus the responder groups [78] A higher number of CD68^+^ TAMs in the chemo-resistant group versus the chemo-sensitive one [68] Increase in CD68^+^, CD163^+^, and CD34^+^ cells in the ABC subgroup versus GCB one [71]
Mantle cell lymphoma	Reduced CD68^+^ and CD163^+^ TAMs in the group with > 40% of Sox11^+^ cells versus negative and 1–39% of positivity groups [72]
Splenic marginal zone lymphoma	Increased number of CD68^+^ and CD163^+^ TAMs in the MALT compared to the healthy ones [75]
MALT lymphoma	Significant increase in CD68^+^ and CD163^+^ TAMs and microvascular density [76]
Follicular lymphoma	Significant increase in CD68^+^ and CD163^+^ TAMs in FL grades versus healthy controls [77]
Classic Hodgkin lymphoma	Significant increase in CD68^+^ and CD163^+^ TAMs and CD34^+^ microvessels in REL patients versus to RESP to ABVC therapy [78]

One of our earliest investigations concerning the microenvironment consisted of counting and mapping out the microvessels in samples of B-NHL. We demonstrated that the stroma of B-NHL has stronger angiogenesis than that of benign lymphadenopathies, and that this angiogenesis intensifies with increase in malignancy grade. Given that B-NHL tumor malignancy grades involve a progression path due to significant increases in tumor cell growth fraction (S-phase and M-phase), this link shows that angiogenesis correlates with tumor progression in these tumor types [67].

We have observed in DLBCL a higher number of CD68^+^ macrophages and microvessels in the non-responder compared to the responder groups [68]. Moreover, we investigated the CD68 expression and its relationship to microvascular density in patients with chemo-resistant and chemo-sensitive DLBCL. A statistically significant greater CD68 expression was seen in the chemo-resistant group respect the chemo-sensitive one. The chemo-resistant group had more vessels counted than patients who were chemo-sensitive [68].

**Fig. 1:**
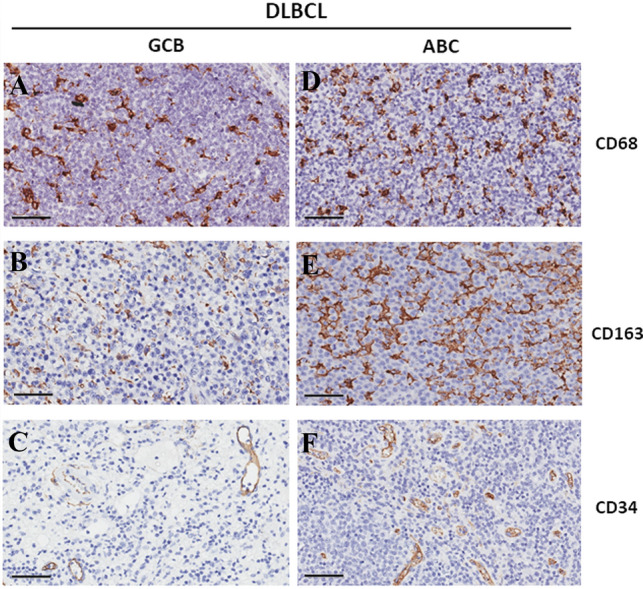
Immunohistochemical staining of CD68, CD163, and C34 in GBC- (**A**–**C**) and ABC-DLBCL (**D**–**F**) samples, scale bar 60 µm

Experimental data in DLBCL suggest that TAMs may contribute to tumor growth by activating STAT3 [69, 70]. We have demonstrated that in the ABC and GCB, DLBCL groups revealed a decrease in CD68^+^, CD163^+^, and CD34^+^ cells in the GBC subgroup (Fig. 1A, B, C) of DLBCL patients respect to ABC one (Fig. 1D, E, F). Additionally, we detected a positive correlation between STAT3 and CD68^+^, CD163^+^ macrophages as well as CD34^+^ vessels [71].

In our research on MCL, we examined the inflammation cell infiltrate in lymph node biopsies taken from patients whose Sox11 expression was either negative, light, or strong. Strong group patients had fewer CD68^+^ and CD163^+^ macrophages than the other two, which was inversely correlated with increased Sox11  intensity and angiogenesis. CD163^+^ cells often outnumbered CD68^+^ cells in each group, and they had both rounded and elongated forms. Our findings demonstrate a mixed M1/M2 population, highlighting the marginal contribution of CD163^+^ rounded and elongated macrophages to the maintenance of a pro-tumorigenic microenvironment in Sox11^+^ patients [72]. Sox11 promotes tumor angiogenesis through transcriptional regulation of PDGF-A [73]. Sox11 is a highly specific marker of MCL because it was detected in around 90% of the MCL examined but in none of the CLL or FL and only weakly in two of 30 DLBCL [74].

In splenic marginal zone lymphoma (MZL), an increased number of CD68^+^ and CD163^+^ macrophages have been demonstrated in the mucosa-associated lymphoid tissue (MALT) compared to the healthy ones [75].

**Fig. 2:**
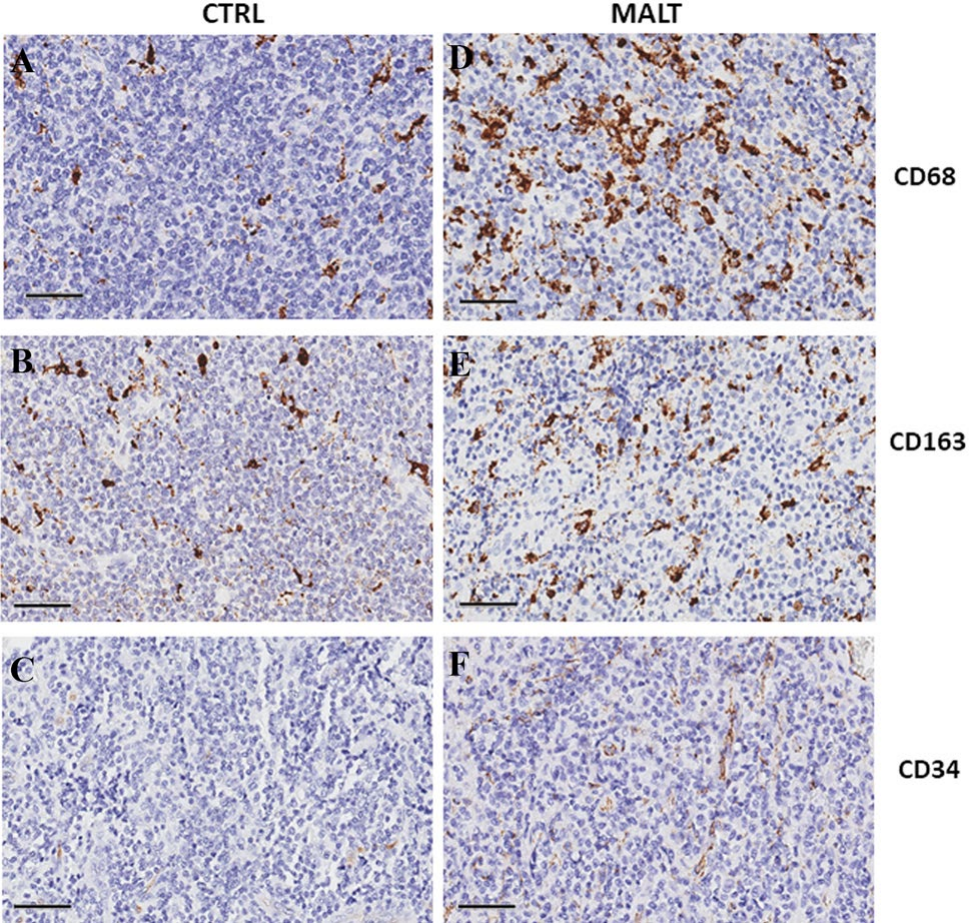
Immunohistochemical staining of CD68, CD163, and C34 in healthy (CTRL) (**A**–**C**) and MALT (**D**–**F**) samples. Scale bar 60 µm

MALT type lymphoma belongs to marginal zone lymphomas (MZL). We examined and quantified the microvessel content and the tumor's inflammatory microenvironment in MALT lymphoma samples and compared them with healthy controls. In comparison with the controls (Fig. 2A, B, C), an increase in CD68^+^ and CD163^+^ TAM numbers as well as microvascular density was seen in the MALT group (Fig. 2D, E, F). Additionally, we discovered a positive correlation between CD34^+^ microvessels and M2 type macrophages, supporting the significance of these cells in angiogenesis [76].

**Fig. 3:**
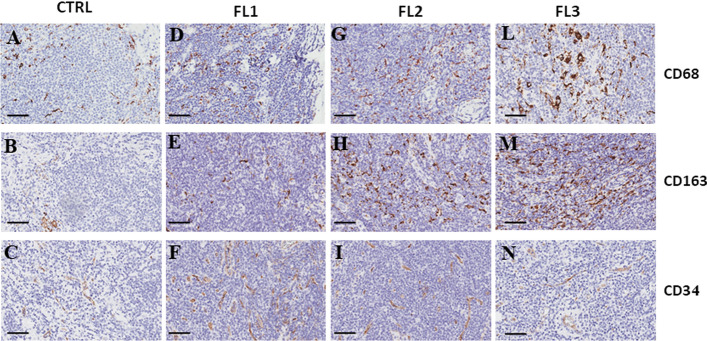
Immunohistochemical staining of CD68, CD163, and C34 in healthy (**A**–**C**) and FL at grades 1 (FL1) (**D**–**F**), 2 (FL2) (**G**–**I**), and 3A (FL3) (**L**–**N**) samples. Scale bar 60 µm

We published the data about the study of inflammatory infiltrate and angiogenesis in lymph node biopsies derived from FL patients at grades 1, 2, and 3A, at first diagnosis and in control healthy group. The results indicated a significant increase in the number of CD68^+^ (Fig. 3D, G, L) and CD163^+^ (Fig. 3E, H, M) macrophages in all three analyzed FL grades respect to the healthy controls (Fig. 3A, B). The number of CD34^+^ microvessels resulted increased in FL1 and FL2 patients (Fig. 3F,I) respect to controls (Fig. 3C) and FL3 patients (Fig. 3N). Moreover, the higher number of CD34^+^ microvessels in the FL grades 1 and 2 of samples positively correlated with CD68^+^ and CD163^+^ cells [77].

**Fig. 4:**
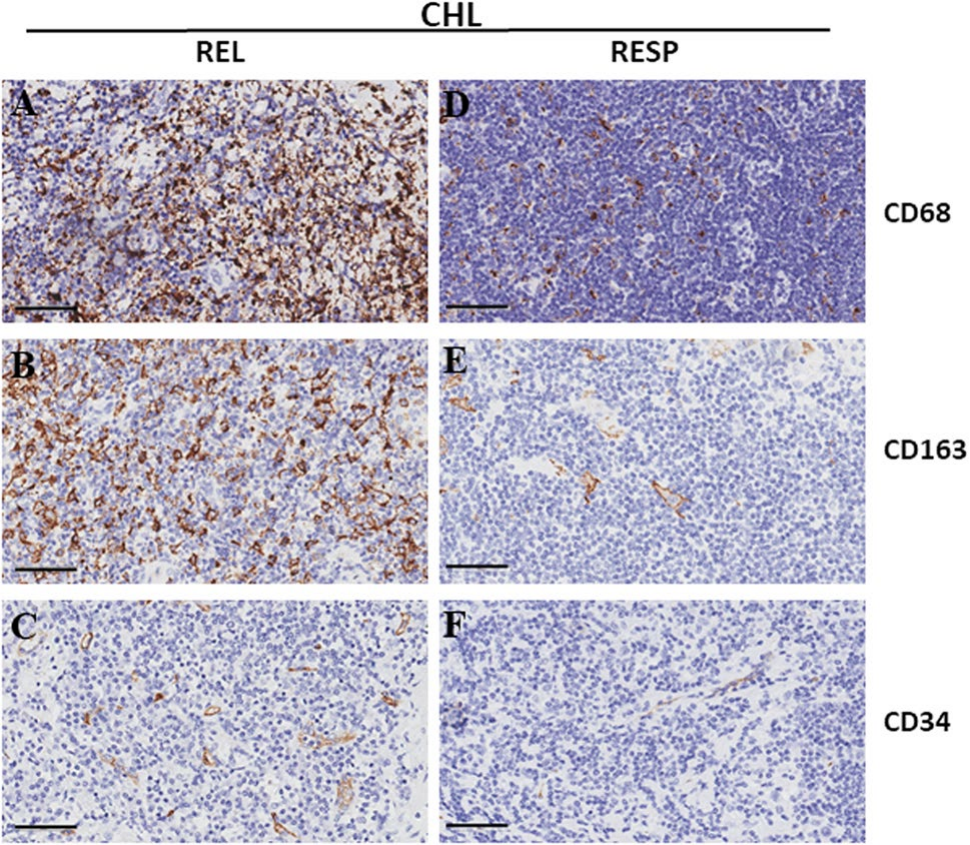
Immunohistochemical staining of CD68, CD163 and C34 in CHL REL (**A**–**C**) and RESP (**D**–**F**) samples. Scale bar 60 µm

The interaction between RS cells and other immune cells in the CHL microenvironment may offer new approaches for targeted immunotherapies. The microenvironment content in CHL patients with ABVD responsive disease (RESP) and patients with disease that had relapsed or was resistant to ABVD treatment (REL) was examined in our most recent research. The findings showed that there were more CD68^+^ and CD163^+^ macrophages and CD 34^+^ microvessels in REL patients (Fig. 4A, B, C) compared to RESP patients (Fig. 4D, E, F), suggesting that the immunological escape in refractory CHL patients involved a greater number of macrophages. Additionally, the findings suggested that microvascular density could be used to evaluate angiogenic activity and aggressiveness in NHL subtypes [78].

## Different therapeutic approaches in lymphomas involving an inhibition of the vascular growth

The major classes of anti-angiogenic therapy include (i) anti-VEGF (bevacizumab, VEGF-Trap, VEGF-antisense); (ii) receptor tyrosine kinase inhibitors targeting VEGF receptor signaling as well as receptors for other pro-angiogenic factors; (iii) immunomodulatory drugs (iMiDs) with anti-angiogenic properties; (iv) the novel anti-endothelial approach of metronomic therapy; and (v) other new compounds targeting signaling checkpoints downstream of pro-angiogenic growth factors, which include mammalian target of rapamycin (mTOR) inhibitors, histone deacetylases (HDAC) inhibitors, and proteasome inhibitors.

Bevacizumab has shown modest clinical activity in lymphoma patients as a single agent in the setting of relapsed aggressive NHL [79] and has been combined with rituximab-CHOP (R-CHOP) in upfront treatment [80]. Levine reported a phase I study of antisense oligonucleotide against VEGF-A in a small cohort of patients and observed a partial response in one patient with cutaneous T cell lymphoma [81]. Bevacizumab synergizes with the BCL2 inhibitor venetoclax in the treatment of B cell NHL [82].

Thalidomide is an-immunomodulatory drug by co-stimulating T cell proliferation which exerts an anti-angiogenic activity through the inhibition of various cytokines, including tumor necrosis factor alpha (TNF-*α)*) and VEGF [83].

Single-agent thalidomide demonstrated a limited and modest overall response rate of 12.5% when given to patients with relapsed/refractory indolent NHL [84].

The anti-angiogenic effects of chemotherapy seem to be optimized by administering such drugs metronomically in small doses on a frequent schedule in an uninterrupted manner, for prolonged periods. A combination of rituximab, thalidomide, and metronomic oral chemotherapy with prednisone, etoposide, procarbazine, and cyclophosphamide have been used as anti-angiogenic therapy in relapsed/refractory MCL [85]. Single-agent lenalidomide has been studied in a phase II trial setting in relapsed/refractory indolent and aggressive NHL, with a 34% overall response rate (ORR) rate in aggressive NHL and a 26% ORR rate in indolent NHL [86, 87].

Metronomic therapy in lymphoma incorporates non-myelosuppressive continuous infusional vincristine and bleomycin designed to overcome drug resistance [88]. Another metronomic lymphoma therapy is the PEPC (C3) regimen [89]. PEPC consists of low-dose prednisone, etoposide, procarbazine, and cyclophosphamide administered orally with dosing frequency titrated to hematologic parameters. This regimen is well tolerated and is associated with significant clinical activity in recurrent NHL including MCL [90, 91]. Recombinant human endostatin has been combined with a CHOP regimen in treating peripheral T cell lymphoma [92]. Treatment with the immunomodulatory lenalidomide, depleted VEGF-C expressing TAMs resulted in impaired lymphangiogenesis in MCL [93].

The limitations of applying bevacizumab in lymphoma treatment may be the consequence of drug resistance, metastasis promotion, and reduced delivery of chemotherapeutic agents, resulted from the decrease in tumor vasculature. The fact that tumors may grow without angiogenesis, through the alternative mode of vasculature neo-formation, including vascular co-potion, intussusceptive microvascular growth, and vasculogenic mimicry [94] makes them less likely to respond to anti-angiogenic drugs. Alternative therapeutic strategies may be used to overcome resistance to anti-angiogenic therapy, including the association of multiple anti-angiogenic compounds or a combination of anti-angiogenic drugs with other treatment regimens. The effectiveness of the combination therapy should be monitored during disease progression to optimize the therapy and counteract the development of further resistance.

## Concluding remarks

There is clear evidence that macrophages play an active role in enhancing angiogenesis in human lymphomas, either directly through the release of angiogenic cytokines and proteolytic enzymes, or indirectly through paracrine signals. A significant relationship between the number of TAMs and the density of blood vessels has been established in human tumors. Depletion of TAMs reduces to about 50% tumor vascular density, leading to areas of necrosis by loss of blood supply within the tumor mass, and macrophages accumulate particularly in such necrotic and hypoxic areas in different tumors. Therapeutic strategies may include inhibition of recruitment of mast cells and macrophages to the tumor microenvironment and blockade of pro-tumoral effects and pro-angiogenic functions [5].
